# Relationships of Sun-Protection Habit Strength with Sunscreen Use During Outdoor Sport and Physical Activity

**DOI:** 10.3390/ijerph9030916

**Published:** 2012-03-15

**Authors:** Sheleigh Lawler, Liane McDermott, David O’Riordan, Kym Spathonis, Elizabeth Eakin, Evie Leslie, Cindy Gallois, Nadine Berndt, Neville Owen

**Affiliations:** 1 Cancer Prevention Research Centre, School of Population Health, The University of Queensland, Herston QLD 4006, Australia; Email: lj_mcdermott@yahoo.com.au (L.M.); k.spathonis@sph.uq.edu.au (K.S.); e.eakin@sph.uq.edu.au (E.E.); 2 Division of Hospital Medicine, University of California, San Francisco, UCSF Medical Center at Parnassus, 505 Parnassus Ave, San Francisco, CA 94122, USA; Email: doriordan@medicine.ucsf.edu; 3 Nutrition and Dietetics, Flinders University, Mark Oliphant Building, G.P.O. Box 2100, Adelaide 5001, South Australia; Email: evie.leslie@flinders.edu.au; 4 Faculty of Social and Behavioural Sciences, The University of Queensland, Brisbane QLD 4072, Australia; Email: c.gallois@uq.edu.au; 5 Department of Psychology, Open University of the Netherlands, P.O. Box 2960, 6401 DL Heerlen, The Netherlands; Email: nadine.berndt@ou.nl; 6 Behavioural Epidemiology, Baker IDI Heart and Diabetes Institute, Melbourne VIC 3004, Australia; Email: Neville.Owen@bakeridi.edu.au

**Keywords:** health behavior, public health practice, sun protection habit strength, outdoor physical activity, sunscreen use

## Abstract

The objective of this cross-sectional questionnaire study was to assess associations of a self-report index of sun protection habit strength with sunscreen use in sporting environments and outdoor physical activity. Participants (n = 234) in field hockey, soccer, tennis and surf sports in Queensland, Australia, completed a self-administered survey on sun protection during organized sport, and during general outdoor physical activity during 2005/2006. The sun protection habit strength index was dichotomized into two categories. Multinomial logistic regression analyses assessed the associations of low *versus* high sun protection habit strength with three categories of sunscreen use (no or rare use; inadequate use; and adequate use). Compared to participants with low sun protection habit strength, those with high sun protection habit strength had significantly greater odds of any sunscreen use during organized sport and during general outdoor physical activity. This association was strongest for adequate sunscreen use in both settings. In conclusion, this study suggests that the measure of sun protection habit strength is a potentially useful assessment tool for future sun protection studies.

## 1. Introduction

Cancer prevention policies and programs have a longstanding focus on sun protection and recently have begun to address physical activity [1]. There are constraints on sun protection practices in most outdoor physical activity settings. For instance, in many sports, safety regulations may prohibit the wearing of hats and sunglasses, and sporting uniforms may impede the protection of all body parts [2]. In such settings, sunscreen use may be the most amenable sun-protective behavior to address, as it is under a high level of volitional control [3,4].

Young adults are more likely to be involved in organized sport compared to any other age group [5], making them a high priority for sport-related sun protection initiatives. In Australia, young adults have had consistent, life-long exposure to summer-time sun protection messages in schools, outdoor environments and the mass media [6]. Thus, it might be expected that they would have internalized such messages, and that prudent sun protection practices would be habitual. However, while intentional sunbathing has decreased in this age group, there is still scope to improve sun protection, as studies have found low-to-moderate levels of sun-protective behavior in young Australian adults [2,6].

In this context, understanding the role of habitual sun protection practices is a key issue for cancer prevention, particularly for active young adults. Habit may be conceptualised as learned sequences of acts that have become automatic responses to specific situational cues [7]. Habit develops by repeating behaviors in stable contexts [8,9]. Verplanken and colleagues’ Self-Report Habit Index has been found to be related to particular health behaviors, including snacking, fruit consumption by children, exercise behavior and television viewing [8,10,11,12], however it has not been used before for sunscreen use. 

We developed a sun protection habit strength index based on Verplanken’s and colleagues habit index and assessed its associations with sunscreen use among young adults, during participation in outdoor organized sport and other outdoor physical activity. Sun protection practices are typically a high priority in organized sports [13,14]. We examined the associations between sun protection habit strength and sunscreen use in two key physical activity behavioral settings (outdoor organized sport and outdoor physical activity).

## 2. Method

Participants were recruited during the summer of 2005/2006 from clubs of four major Australian outdoor sports: field hockey, soccer, tennis and surf sports. Twenty-four clubs of those eligible (56%) agreed to participate. Competitors from these sporting clubs (aged 18–30 years who participated in their sport weekly) were asked to complete a self-administered survey. When possible, survey packages were distributed to and collected prior to or after a competition or training session by research assistants. When this was not possible, coaches distributed the surveys to players in person, or club officials mailed the surveys out on behalf of the study, with a reply-paid envelope. Detailed methods of recruitment have been described elsewhere [2]. Ethics approval was obtained from the University of Queensland’s Behavioral and Social Sciences Ethical Review Committee. 

### 2.1. Measures

#### 2.1.1. Outcome Variables: Sunscreen Use

Two outcome variables were created that assessed sunscreen use: sunscreen use in organized sport settings and sunscreen use in general outdoor physical activity. *Sunscreen use in organized sport settings* was established from respondents’ self-reported sunscreen use during their most recent game played on a sunny day (yes/no), the sun protection factor (SPF) used (<30+ or ≥30+) and reapplication of sunscreen during the game (yes/no). A SPF of ≥30 was used as the reference as this is in line with Australian skin cancer prevention guidelines. These questions were used to derive three categories for organized sport: no sunscreen use; inadequate use of sunscreen (used sunscreen but SPF <30+ and/or sunscreen not reapplied); and, adequate use of sunscreen (used SPF ≥30+ sunscreen and sunscreen reapplied). *Sunscreen use during general outdoor physical activity* (between the hours of 10 am and 2 pm) was assessed with response categories of ‘never’, ‘very rarely’, ‘rarely’, ‘occasionally’, ‘very frequently’, and ‘always’. Three categories were derived: no/rare sunscreen use; inadequate use of sunscreen (occasionally); and, adequate use of sunscreen (frequently/always).

#### 2.1.2. Explanatory Variables

Habit strength was the main explanatory variable of interest. A shortened 6-item version of the original 12-item Self-Report Habit Index [10] was used, based on pilot studies to examine the face validity of potentially relevant items for the context of sun-protective behaviors in sport and physical activity settings. Participants were asked to rate on a 6-point Likert scale (ranging from ‘agree very strongly’ to ‘disagree very strongly’), the extent to which they agreed or disagreed with the following six items: “Protecting my skin from the sun whenever I am outdoors being physically active is something: (1) I do frequently; (2) I do without thinking; (3) that belongs to my (daily, weekly, monthly) routine; (4) I start doing before I realize that I’m doing it; (5) that’s typically me; (6) I have been doing for a long time”. The responses to the six items were summed to create an index that ranged from 6 to 36. Based on a median split, 24 and above was considered as higher habit strength; below 24 was considered to be lower habit strength. Factor analysis showed that all six items loaded onto one factor (item loading scores ranged from 0.71–0.85), which is consistent with the original Self-Report Habit Index [10]. Internal consistency was high (Cronbach’s alpha = 0.95). 

Other explanatory variables included were gender, skin type and history of sunburn. A skin type index was created from two self-reported questions: one assessing skin colour (responses ranging from ‘very fair’ to ‘dark or black’), and the other assessing skin reaction to UV-radiation when being exposed to one hour of midday sun (responses ranging from ‘burns easily, never tans’ to ‘never burns, tans profusely’). The scores for both items were summed, providing a continuous score from 1 to 11. Four skin types were identified: ‘very fair skin, burns easily’, ‘fair/medium skin, moderately burns’, ‘olive skin, rarely burns’ and ‘dark/black skin, never burns’ [15,16]. Two skin type categories were derived: hardly burns and easily burns. History of sunburn was assessed by asking “since the beginning of your last sporting season, has any part of your body been sunburned while participating in your sport? (yes/no)”.

### 2.2. Statistical Analyses

Two multinomial logistic regression analyses were performed using sunscreen use while participating in sport and during general outdoor physical activity as the main outcome measures. Habit strength was used as the main explanatory variable in both multinomial logistic regression analyses. No or rare use of sunscreen was the referent category, and the odds of having inadequate or adequate levels of sunscreen use while participating in sport and during general outdoor physical activity were examined. All analyses were adjusted for gender, skin type and history of sunburn and were conducted using SPSS version 13.0 for Windows. Significance was accepted at the 0.05 level.

## 3. Results

The survey was completed by 237 participants (40.9% men and 59.1% women), with a mean age of 23.2 years (SD 3.8). For the organized sport setting, 29.5% of participants reported no sunscreen use, 47.7% inadequate use and 20.2% adequate use. During general outdoor physical activity, 26.6% reported no or rare sunscreen use, 24.1% inadequate use and 48.9% adequate use of sunscreen.

Table 1 shows the multinomial logistic regression model evaluating the odds of using sunscreen compared to no sunscreen use during organized sport; Table 2 shows the multinomial logistic regression model for no or rare sunscreen use during general outdoor physical activity. High sun protection habit strength was significantly associated with sunscreen use in both settings. This association was strongest for adequate sunscreen use: participants with high sun protection habit strength had an odds ratio of adequate sunscreen use 17 times that of those with low habit strength during organized sport and 24 times that of those with low habit strength during general outdoor physical activity. A history of sunburn was also consistently associated with sunscreen use in both sporting settings.

**Table 1:** Odds ratio (95% CI) of inadequate and adequate sunscreen use compared to no sunscreen use during organized sport.

Organized Sport			
	*(n)*	*Inadequate sunscreen use* (n = 113)	*Adequate sunscreen use* (n = 48)
**Gender**			
Men	(94)	1.00	1.00
Women	(140)	3.27 (1.62–6.63) ***	1.33 (0.53–3.37)
**Skin type**			
Hardly burns	(97)	1.00	1.00
Easily burns	(137)	1.35 (0.67–2.73)	1.82 (0.71–4.69)
**History of sunburn**			
Not sunburned	(66)	1.00	1.00
Sunburned	(149)	2.74 (1.32–5.68) **	6.97 (2.19–22.21) ***
**Habit strength**			
Low	(133)	1.00	1.00
High	(103)	2.98 (1.33–6.67) ***	17.38 (6.21–48.64) ***

NB: The wide confidence intervals for high habit strength relate to low expected frequencies in the no sunscreen use referent group. * *p* ≤ 0.05; ** *p* ≤ 0.01; *** *p* ≤ 0.001.

**Table 2:** Odds ratio (95% CI) of inadequate and adequate sunscreen use compared to no or rare sunscreen use during general outdoor physical activity.

Outdoor Physical Activity			
	*(n)*	*Inadequate sunscreen use* (n = 57)	*Adequate sunscreen use* (n = 116)
**Gender**			
Men	(94)	1.00	1.00
Women	(140)	1.04 (0.47–2.27)	1.97 (0.88–4.43)
**Skin type**			
Hardly burns	(97)	1.00	1.00
Easily burns	(137)	1.20 (0.54–2.63)	2.37 (1.05–5.35) *
**History of sunburn**			
Not sunburned	(66)	1.00	1.00
Sunburned	(149)	2.44 (1.05–5.67) *	1.97 (0.85–4.59)
**Habit strength**			
Low	(133)	1.00	1.00
High	(103)	2.96 (0.94–9.33)	24.69 (8.79–69.41) ***

NB: The wide confidence intervals for high habit strength relate to low expected frequencies in the no or rare sunscreen use referent group. * *p* ≤ 0.05; ** *p* ≤ 0.01; *** *p* ≤ 0.001.

## 4. Discussion

Among the young Australian sport participants in this study, sun protection habit strength was strongly associated with sunscreen use, both during organized sport and during general outdoor physical activity. Our short sun protection habit strength measure, based on the Self-Report Habit Index [10], discriminated clearly between sunscreen use and non-use in two different outdoor physical activity settings. Furthermore, the potential role of habit strength appeared particularly important for more vigilant use of sunscreen (*i.e.*, SPF ≥30+ and reapplication, and frequently/always use sunscreen). Our results show that there are stronger associations between habit strength and sunscreen use in outdoor physical activity settings than for organized sports settings. Furthermore, there are associations between high habit strength and inadequate sunscreen use in organized sport. This may be due to sun–protection constraints often present in organized sports settings, for example, sunscreen getting in eyes and making hands slippery. Interestingly, there were gender differences in sunscreen use in organized sport, but not outdoor physical activity, with women compared to men having inadequate sunscreen use, which may be due to club social norms. Sun protection habits of young adults still need to be improved, both in sport-specific contexts and during general outdoor physical activity. Support could be provided in the form of policies and regulations which facilitate the provision of sunscreen and other sun-protection behaviors and are tailored to individual sport settings [2,17], while more focussed skin cancer prevention campaigns are needed to improve sun protection habit strength in active young adults. 

## 5. Study Limitations

The study included few different sports and recruited participants via convenience. Coupled with the lack of information on non-participants and inability to assess response rates, representativeness and generalizability may be limited. More women than men participated in the study despite more men than women in Australia participating in organized sports [18] and we do not know whether sun-protection habits and practices in the sample are reflective of the general public. Temporality and causality cannot be determined. Furthermore, a limited range of sunscreen-related attributes were assessed: other personal, social-cognitive and environmental factors should be included in future studies.

## 6. Conclusions

Future research could explore the role of sun protection habit in other contexts, such as at the beach, on holidays and other aquatic, trail use or bicycle riding settings. This brief measure of sun protection habit strength is a potentially useful assessment tool for such studies. Furthermore, skin cancer prevention campaigns could use this measure to assess whether sun protection behaviors are shifting from reflective to habitual. As a multi-item index, it may be more responsive to change in the context of evaluating the effectiveness of prevention programs and campaigns, although this will need to be determined in future investigations.
